# Farming the Sea: The Only Way to Meet Humanity's Future Food Needs

**DOI:** 10.1029/2019GH000204

**Published:** 2019-09-09

**Authors:** Jerry R. Schubel, Kimberly Thompson

**Affiliations:** ^1^ Aquarium of the Pacific Long Beach CA USA

**Keywords:** aquaculture, agriculture, sustainable food systems, food and climate change, farming, food supply

## Abstract

A major change began 10,000–12,000 years ago when humans began to practice agriculture. A series of “green revolutions” enabled the human population to explode, but these advancements have dramatically changed the planet. The United Nations predicts that we will need to produce 50% more food by 2050 to feed another 2.5 billion people, but this will be challenging with tighter land and water resources and a changing climate. Responsible marine aquaculture can complement responsible land‐based agriculture and aquaculture and well‐managed fisheries to increase the global supply of nutritious food.

## Key Points

- Agricultural innovations have facilitated an explosion in human population growth and dramatically changed the planet
- The capacity for land‐based agriculture to mitigate ecosystem impacts at scale is limited
- We need to integrate agriculture with aquaculture, including ocean‐based farming, to meet the food needs of our growing population

## 1 Humans and Food

Our species, *Homo sapiens*, dates back 200,000–300,000 years. For more than 95% of our history, we were hunter‐gatherers, living in small groups and wandering across the landscape with the seasons in search of food, primarily fruits and nuts but also terrestrial animals and seafood (Crittenden & Schnorr, 2016). Our population was limited by food supply.

A major change began 10,000–12,000 years ago when humans began to practice agriculture. With a predictable food supply, people could settle down into villages. Over time, the villages developed into towns and eventually into cities. The innovations in agriculture facilitated an explosion in human population growth. Through a series of “green revolutions” we avoided the mass starvation predicted by Malthus, Paul Ehrlich, and others, but these advancements have dramatically changed the planet (Pingali, 2012; Trewavas, 2002).

As the population and the demand for food grows, so does agriculture's toll on Earth and the ocean. Today, global agriculture consumes 70% of all available freshwater and almost half of Earth's ice‐free land surface (FAO, 2017a; Hooke et al., 2012). It also accounts for one third of all greenhouse gas emissions, with livestock contributing nearly half of this total. In addition, runoff from terrestrial agriculture contributes to the growing prevalence and frequency of dead zones in coastal waters and negatively impacts local marine ecosystems and species.

The United Nations predicts that we will need to produce 50% more food by 2050 to feed another 2.5 billion people (FAO, 2017b). Present agricultural practices cannot scale to meet this demand without a major modification in the way agriculture is implemented. There are not enough freshwater or land resources, and environmental burdens (greenhouse gas emissions, runoff, and soil degradation) will only increase (Duarte et al., 2009; FAO, 2017a; Willett et al., 2019).

The uncertainty and variability that result from climate change makes producing 50% more food challenging. Changes in temperature and precipitation regimes will produce agricultural winners and losers, but on balance global agriculture yields are likely to decrease. A meta‐analysis of more than 1,000 studies on agricultural yields under different climate conditions found that climate change may significantly reduce yields in the long run (Porter et al., 2014). One factor that plays a large role is the decreasing availability of and access to freshwater. Predicted conflicts over this life‐essential resource will impact future agricultural yields, including livestock, in many parts of the world (Duarte et al., 2009; FAO, 2017b).

Livestock, particularly cattle, is an important and growing sector in agriculture. Demand for animal protein in the form of meat, milk, and eggs is increasing. Some factors contributing to this increase in consumption include the growing global population and increasing incomes. The production of these animal protein sources takes a large environmental toll. Raising livestock requires a significant amount of freshwater and land both to support animals directly and to produce the agricultural crops they require as feed. Livestock production also accounts for between 14.5% and 18% of human‐induced greenhouse gas emissions (FAO, 2017b; Searchinger et al., 2018). Most of these emissions are directly emitted by cattle and other ruminants, like sheep and buffalo, which use bacteria in their stomachs to ferment food. The result is the release of methane into the atmosphere when these animals burp (FAO, 2017b).

There are a number of innovations on the horizon that can increase the production of nutritious food from responsible agriculture while minimizing environmental impacts, including precision farming, permaculture, and advancing genetic selection technologies such as GMOs and CRISPR (FAO, 2017b). These innovations will play an important role in creating a sustainable and more climate‐resilient food portfolio, but their capacities to mitigate ecosystem impacts at scale are highly dependent upon changes and transformations at societal and governance levels that may take more time to achieve. In the meantime, countries that have the capacity to increase marine aquaculture responsibly can support much needed changes by integrating responsible land‐based agriculture and aquaculture with ocean‐based aquaculture to meet the food needs of our growing population.

## 2 Can The Ocean Save Us? It May Have To

The ocean has been an important source of protein for thousands of years. It may even have saved our species from extinction. More than 70,000 years ago, a volcano called Toba erupted in Indonesia. Some contend that it spewed so much ash that the Earth may have gone into the equivalent of a nuclear winter for 6 to 10 years, although this has not been well established (Rampino & Self, 1992; Yost et al., 2018). Temperatures plummeted, and much of life on Earth was extinguished. Some anthropologists contend that *Homo sapiens* almost went extinct around the same time that Toba erupted as the human population was reduced to only a few thousand breeding pairs (Rampino & Ambrose, 2000). Many lived in caves near Pinnacle Point on the east coast of South Africa, and survived by eating shellfish and finfish. Based on these findings, some scientists contend that the ocean—and seafood—played a role in saving our species from extinction (Marean, 2010, 2012; Marean et al., 2007).

Today, the ocean produces about 17% of the world's per capita consumption of animal protein (FAO, 2018a; Bennett et al., 2018). The ocean's contribution to the total global food supply is far less impressive. While covering 71% of Earth's surface, the world ocean contributes only 2% to the world's food supply on a caloric basis (FAO, 2018b). Protein from seafood is a significant source of high‐quality nutrients and is essential in the diets of some densely populated countries where protein consumption is low (FAO, 2018b; Bennett et al., 2018). In developed countries, like the United States the overall demand is high, but per capita consumption does not meet recommendations from government and health organizations: to eat at least two servings of approximately four ounces of seafood per week to support heart health. Americans eat less than half the recommended amount United States Department of Health and Human Services (USDOH) & United States Department of Agriculture ((USDA), 2015). Eating more seafood—compared to beef, lamb, and pork—to meet our animal protein requirements would have significant human health and environmental benefits (Willett et al., 2019).

## 3 The Modern Hunter‐Gatherer

It took hundreds of years, but the majority of humans have abandoned their hunter‐gatherer lifestyle, except in the 71% of Earth covered by seawater where the hunter‐gatherer lifestyle persists. We call it fishing, and if left unchecked it threatens to have an effect on marine life similar to what hunter‐gatherers had on terrestrial life. As hunting tools became more sophisticated and the human population grew and expanded, fragmenting and degrading habitat, we drove many of our prey to the brink of extinction and pushed some—like the wooly mammoth, the dodo, the saber tooth tiger, the passenger pigeon, and about 25 other species in North America alone—over the edge (Martin, 1966).

We see ominous signs of what happened on land beginning to happen in the world ocean. To date we have driven a number of ocean species to economic extinction, where their populations are so low it is no longer economically viable to pursue them (Courchamp et al., 2006). In some cases, such as with Atlantic cod, moratoria prohibit or severely restrict harvests in hopes that their populations will rebound and they can once again provide an economically and ecologically sustainable source of food (Hamilton & Butler, 2001). In other cases, we have driven species to ecological extinction, meaning that their populations are so low they no longer play the roles in their ecosystem they once did (Estes et al., 1989). We have even driven a few to biological extinction, meaning they are gone forever (Raup, 1986).

Some countries, including the United States, have established environmentally and economically responsible management of wild‐capture fisheries in their territorial seas. Well‐managed fisheries play an important role in providing nutritious food to the growing and increasingly affluent population (Davis et al., 2016; FAO, 2018b; Hallstrom et al., 2019). They also provide jobs and conserve working waterfronts that are magnets for tourists, but wild‐capture fishing cannot keep pace with the growing demand for seafood. The production from wild seafood harvests have been flat for decades. According to the United Nations Food and Agricultural Organization (FAO), more than 90% of global fish stocks are fished at or above sustainable limits. With appropriate science‐based management that includes setting aside more and larger areas of the ocean for protection, some expansion of wild‐capture harvests is possible, but this expansion will be modest (Bennett et al., 2018; Costello et al., 2016; Edgar et al., 2018; FAO, 2018b; Lester et al., 2009).

Clearly, land‐based agriculture and wild‐capture fisheries cannot provide a consistent supply of food that is scalable to meet the needs of a growing population in a changing climate without increasing environmental impacts. Some forms of aquaculture may also provide ecosystem benefits (Alleway et al., 2018). Aquaculture, or the farming of aquatic organisms, plays a significant role in meeting the growing demand for seafood and already accounts for nearly half of the world's edible seafood supply (FAO, 2018b). Most aquaculture takes place in freshwater systems, which have limited capacity for sustainable growth in an environment where freshwater resources are increasingly limited. Marine aquaculture, or farming the sea, is a promising opportunity to leverage the ocean to complement responsible land‐based agriculture and well‐managed fisheries to increase the global supply of nutritious food with minimal impacts on the ocean and the environment. It must be done using the best and most appropriate science and technology (Lester, Gentry, et al., 2018).

## 4 Moving Food Production to the Sea

The world ocean is the largest single component in Earth's life support system. We rely on it for essential ecosystem services. The delivery of those services depends upon having healthy, productive, and diverse ecosystems. The ocean provides opportunities for expanding the production of food while relieving pressures on wildlife and natural ecosystem functions on land, which are experiencing the fastest rates of extinction because of habitat loss (Cardinale et al., 2012; Diaz et al., 2019; Froehlich et al., 2017; Powers & Jetz, 2019; Waite et al., 2014). Responsibly farming in the sea can benefit society and minimize unfavorable impacts in order to sustain healthy ocean ecosystems (Lester, Gentry, et al., 2018).

Marine aquaculture requires little land and freshwater, most of which is associated with producing feed inputs and processing (Froehlich, Runge, et al., 2018). It results in fewer greenhouse gas emissions compared to land‐based animal agriculture (Davis et al., 2016; Waite et al., 2014). Shellfish and seaweed aquaculture can even provide ecosystem services, such as cleaning the water, stabilizing shorelines, and storing CO_2_ (Alleway et al., 2018). Requirements for terrestrial feed inputs are expected to diminish with improving technology for open‐ocean seaweed production and fermentation. Despite historic reliance on wild fish, the most current feed formulations for fish farms have significantly reduced the fish meal component through replacement with fish wastes, plant material, insects, rendered animal proteins, microbial proteins, and algal oils. These and other innovations provide feed that are healthful to the fish, maintain the desired seafood qualities consumers look for, and reduce the strain of aquaculture production on environmental resources.

This does not mean that farming in the marine environment is not without risks. Events associated with real and perceived risks (e.g., escapes, disease, effluent discharge, and wildlife interactions) can and will happen. While the state of the science for best practices and technologies that can minimize these risks is growing and improving, there is uncertainty and justified concern around the potential long‐term and local effects associated with some of these risks. Using science‐based siting, best practices, collaborative monitoring, and management along with appropriate technologies and farming methods (e.g., integrated multitrophic aquaculture) can substantially reduce the probability of severe, irreversible impacts from these risks, and in some cases, eliminate them altogether (FAO, 2018b; Lester, Stevens, et al., 2018; Price & Morris, 2013; Rust et al., 2014).

Marine aquaculture is also not immune to the impacts of climate change. Shellfish can be severely impacted by the effects of ocean acidification while finfish may be impacted by the changing ocean temperatures, low oxygen levels, and algal blooms. But there is a growing body of research highlighting its potential to contribute to climate resilience (Froehlich, Gentry, & Halpern 2018; Klinger et al., 2017). Seaweed, for example, can absorb CO_2_ and provide a buffer from the effects of ocean acidification on sensitive habitats (e.g., coral reefs) and shellfish (Gentry et al., 2019; Mongin et al., 2016). Shellfish and seaweed have the potential to increase coastal resilience by providing habitat benefits to wildlife and supporting ecosystem services, that is, improved water quality, especially through seaweed aquaculture (Alleway et al., 2018; Gentry et al., 2019). Research also shows that, with the implementation of best management practices, finfish can be more resilient to changing ocean temperatures (Klinger et al., 2017).

## 5 The U.S. Opportunity and Responsibility

The United States is a global leader in seafood sustainability and has some of the most stringent and effective laws to protect the environment and public health in terms of food safety and workers' rights. Despite this, we import more seafood than any other nation (FAO, 2018b). About 60% of our seafood comes from other countries, mostly from Asia, and more than half from farmed sources (Gephart et al., 2019).

As a leading consumer of animal protein products, the United States has an ethical obligation to leverage its economic, regulatory, and scientific resources to produce more of these products domestically. International trade can obscure traceability and thus increase the likelihood for products lacking sufficient environmental, social, and/or food safety standards to enter the market (FAO, 2018b; Lester, Gentry, et al., 2018; National Marine Fisheries Service (NMFS), 2018). The national population of a major source of our seafood imports, China, is increasing and the growth rate of their middle class is rapidly outpacing that of their population (Barton, 2013). They can afford to buy more of the higher‐quality seafood they produce that was historically exported to other wealthy countries. The United States and other countries that are heavily reliant on exports from countries like China will have to find other sources of seafood to meet domestic demand.

Despite having the second largest Exclusive Economic Zone in the world, the United States lags behind the rest of the world in marine aquaculture production (Kapetsky et al., 2013). U.S. marine aquaculture accounts for just 6% of our total domestic food supply and ranks 17th in the world in aquaculture production (FAO, 2018b). As a result, the annual U.S. seafood trade deficit has grown to nearly $14 billion (NMFS, 2018). A UN study concluded that of all nations, the United States has the greatest potential for marine aquaculture based on a number of factors including ecological conditions, markets, and waterfront infrastructure (Kapetsky et al., 2013). Another study concluded that the United States could produce an amount of seafood equivalent to all wild‐capture fisheries landings globally in a surface area of ocean about the size of Lake Michigan (Gentry et al., 2017). This is equivalent to less than 0.1% of the U.S. Exclusive Economic Zone. In contrast, the United States devotes more than 50% of its land surface area to agriculture and most of that area is for livestock (Bigelow & Borchers, 2017). Siting farms offshore provide more opportunities to produce higher‐quality seafood at scale with fewer environmental impacts and conflicts with other ocean users.

But scaling up is easier said than done. Offshore operations will be higher in cost than nearshore and land‐based aquaculture production. Achieving economies of scale will be critical for successful offshore marine aquaculture businesses. This will take time (Packard Foundation, 2018). Siting and operating farms offshore also requires significant investment. It is difficult to secure investments when the process to permit these operations is so unpredictable. Securing permits in state and federal waters is difficult and time consuming. With few exceptions, permitting marine aquaculture requires a lot of time and money that can discourage investments and growth in the sector. In many cases, including permitting in federal waters, this is because the regulatory framework is complex and lacks leadership. In others it is due to the challenges associated with negative public perceptions, typically driven by small but politically influential special interest groups that can stop projects at an early stage. We need a permitting process that is transparent, based on the best science, and has greater predictability without sacrificing regulations and standards that protect the environment.

## 6 The Ocean Has the Potential to Save Us Again

Meeting the fundamental food, water, and energy needs of the growing population in a rapidly changing climate is putting immense pressure on the ocean and land systems. While land‐based ecosystems bear much of the impact from production of these resources, the impacts do not stop at the shoreline. The land and sea are connected. Terrestrial actions can result in irreparable impacts to the shared commodity that is our global ocean. We have an ethical responsibility to ensure that all of the world's citizens have consistent access to safe and secure sources of food, water, and energy, but the ability of land‐based systems to meet these needs is increasingly limited. Marine aquaculture is one of the many tools available in our toolkit to help meet these needs.

Mistakes have been made in aquaculture—some bad mistakes—and they must be acknowledged. Farms have been sited in the wrong places. Cages have been overstocked and degraded the environment. Important coastal habitats, like mangroves, have been destroyed to make room for shrimp farms. Aquaculture has sometimes contributed to the decline of fisheries for small fish, especially in the multispecies tropical fisheries of Asia, to feed the fish growing in farms. As with any form of production to provide food, water, and energy to the growing population, the risks are not zero, but the lessons learned from past mistakes and advances in scientific knowledge and technological development provide a promising opportunity to grow marine aquaculture responsibly to produce more nutritious food with fewer environmental impacts.

The environmental and human health benefits of farming more of our animal protein in the ocean are scientifically clear. More and more, large‐scale data synthesis research is pointing to important role a diverse marine aquaculture sector (finfish, shellfish, and seaweed) can and should play in a sustainable, more climate‐resilient food supply (Davis et al., 2016; Froehlich, Gentry, & Halpern 2018; Froehlich, Runge, et al., 2018; Searchinger et al., 2018). The state of the science is strong for best practices and appropriate technologies to increase production efficiencies while reducing impacts from marine aquaculture, ensuring that it is done right. Significant advances have been made in our knowledge about how to farm responsibly. It is imperative that investments continue to be made to advance scientific knowledge and technological capacities to ensure that we can produce the food that is needed to feed people, while protecting our ocean environment. Marine aquaculture provides promising opportunities to increase our supply of nutritious food, while using fewer land and freshwater resources and supporting efforts to protect biodiversity on land. It can also contribute to a more climate‐resilient food supply and can provide ecosystem benefits by cleaning the water, providing habitat, and stabilizing shorelines.

Marine aquaculture can play an important role in the story of our future: a sustainable food future where everyone has access to a consistent supply of safe, secure, and nutritious food in a changing climate.

### Conflict of Interest

The authors declare no conflicts of interest relevant to this study.
